# Protocol for extramedullary hematopoiesis in patients with transfusion‐dependent β‐thalassemia (TDT): A systematic review

**DOI:** 10.1002/hsr2.429

**Published:** 2021-11-03

**Authors:** Fateen Ata, Eihab A. Subahi, Hassan Choudry, Mohamed A. Yassin

**Affiliations:** ^1^ Department of Internal Medicine Hamad General Hospital, Hamad Medical Corporation Doha Qatar; ^2^ Department of Internal Medicine Punjab Medical College Faisalabad Pakistan; ^3^ Department of Hematology National Centre for Cancer Care and Research, Hamad Medical Corporation Doha Qatar

**Keywords:** beta‐thalassemia major, extramedullary hematopoiesis, non‐medullary hematopoiesis, transfusion‐dependent thalassemia

## Abstract

**Background and aims:**

Thalassemia is one of the most common hemoglobinopathies, with around 5% of the world's population expected to have some degree and type of thalassemia. Beta thalassemia (BT) occurs due to a deficient production of the beta‐globin chain of hemoglobin. Extramedullary hematopoiesis (EMH) is one of the complications of BT, mainly observed in minor/intermedia subtypes. EMH is the production of blood cells outside the marrow as a compensatory response to longstanding hypoxia. Due to chronic transfusions, it is not expected in patients with beta‐thalassemia major (BTM). However, there are increasingly reported cases of EMH in BTM. The incidence of EMH in BTM is thought to be <1%. However, it seems that the true incidence is much higher than expected. This review aims to pool the available data and provide cumulative evidence on the reports of EMH in BTM patients.

**Methods:**

We aim to conduct a systematic review via searching multiple electronic databases (PubMed, Scopus, Google Scholar) to identify eligible articles from any date up to December 2020. Eligible studies should report extramedullary hematopoiesis in BTM. Case reports, case series, observational studies with cross‐sectional or prospective research design, case‐control studies, and experimental studies will be included if found relevant. Two reviewers (FA and ES) will individually analyze the study quality using the statistical methodology and categories guided by the Cochrane Collaboration Handbook, PRISMA guidelines, and Joanna Briggs Institute checklist for case reports and series.

**Results:**

This study will analyze and incorporate the available evidence on EMH in BTM concerning patient demographics, sites of EMH, management, and clinical outcomes of EMH.

**Conclusion:**

By summarizing and statistically analyzing the data about EMH in BTM, this study will generate extensive knowledge on the topic for a better understanding of atypical presentations in BTM, a common hemoglobinopathy.

## Graphical abstract

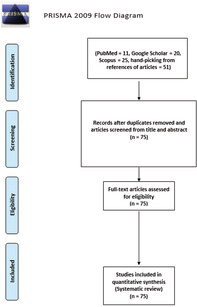

**Abbreviations**
BTM: Beta‐thalassemia major
EMH: Extramedullary hematopoiesis
JBI: Joanna Briggs Institute
MOOSE: Meta‐analyses of Observational Studies in Epidemiology
PRISMA: Preferred Reporting Items for Systematic Reviews and Meta‐Analyses
PRISMA‐P: Preferred Reporting Items for Systematic Reviews and Meta‐Analyses for protocols
PROSPERO: International Prospective Register of Systematic Reviews
RCT: Randomized controlled trial
TDM: Transfusion‐dependent thalassemia

## 1 INTRODUCTION

Thalassemia is a type of hypochromic microcytic anemia caused by a lack of or decreased synthesis of hemoglobin's globin chain.^1^ One of its main subtypes is beta‐thalassemia, which occurs as a consequence of mutation of the beta‐globin gene, resulting in a decreased production of the beta‐globin chain.^2^ Generally, beta‐thalassemia can be divided into two subtypes: transfusion‐dependent thalassemia (TDT) and non‐transfusion‐dependent thalassemia (NTDT).^3^ TDT consists mainly of beta‐thalassemia major (BTM), and NTDT comprises beta‐thalassemia minor and beta‐thalassemia intermedia.^3^ Depending upon the severity of the thalassemia, patients can present with only incidental findings of anemia, to significant clinical manifestations such as growth retardation, recurrent infections, hepatosplenomegaly, and heart failure.^4^ In suspected patients (identified by incidental findings, screening programs, manifestations of thalassemia), a diagnosis can be made by peripheral blood film, hemoglobin electrophoresis, and gene analysis.^3^ Treatment for NTDT mainly comprises symptom alleviation with infrequent blood transfusions, hemoglobin F augmentation, iron chelation, and splenectomy if needed. On the other hand, treatment of TDT requires regular blood transfusions with iron chelation therapies and curative management via stem cell transplantation.^3^ With the advent of gene therapy, there is a promising future in the curative management of BTM.^5^

One of the complications of beta‐thalassemia includes EMH. EMH is a condition in which blood production occurs outside the physiological domain, the bone marrow. EMH can occur in various organs, including the spleen, liver, lymph nodes, thymus, heart, breasts, prostate, kidneys, adrenal glands, pleura, retroperitoneal tissue, skin, peripheral and cranial nerves, and the spinal cord.^6^ EMH is more common in NTDT (20%) compared with TDT (1%).^7^ However, the prevalence of EMH as high as around 13% has been reported in TDT patients in one study.^8^ Consequently, it has been studied in NTDT more extensively compared with TDT, in which data are limited mainly to case reports.^9^ Due to its rare occurrence, data related to EMH in TDT are scattered and need compilation to provide cumulative evidence on its demographics, clinical course, and more so, its management and outcomes. There is at least one retrospective study and multiple case reports on EMH in BTM with variable management strategies and outcomes.^6^, ^7^, ^8^, ^10^ We have conducted a thorough literature search and identified a total of 254 patients with TDT and EMH. This systematic review will be among the first ones to provide an aggregate evidence on demographics, management, and outcomes of EMH in TDT.

## 2 METHODS

### 2.1 Registration

The protocol is registered at PROSPERO: CRD42021242943.

### 2.2 Inclusion and exclusion criteria

#### 2.2.1 Types of studies

Eligible studies (English language) should report EMH in BTM. All articles, including clinical trials, case reports, case series, and observational studies (retrospective and prospective) from any date till December 2020, will be included. Studies in languages other than English will not be included.

#### 2.2.2 Participants

The study population will comprise the population of any age (adults and pediatrics) who had EMH with a diagnosis of BTM. The included patients should have a confirmed diagnosis of BTM and should have a documented evidence of EMH diagnosed by imaging ± biopsy.

Exclusion criteria include patients with beta‐thalassemia variant other than major (trait, minor, intermediate) of patients with BTM but no evidence of EMH.

### 2.3 Search terms and search strategy

We will follow MOOSE Guidelines for Meta‐Analyses and Systematic Reviews of Observational Studies and the PRISMA‐P (Preferred Reporting Items for Systematic Reviews and Meta‐Analyses) guidelines to conduct this review and the protocol.^11^, ^12^, ^13^

Systematic searches will be performed in the following electronic databases: PubMed, Scopus, and Google scholar to identify eligible articles to achieve the study objectives. The following search term will be used “beta‐thalassemia major” OR “transfusion‐dependent thalassemia” OR “TDT” and “Extramedullary hematopoiesis” OR “non‐medullary hematopoiesis.” The search results from the above databases will be downloaded into the citation manager, where duplicates will be removed, and phase‐wise screening will be performed.

### 2.4 Study selection

**FIGURE 1:**
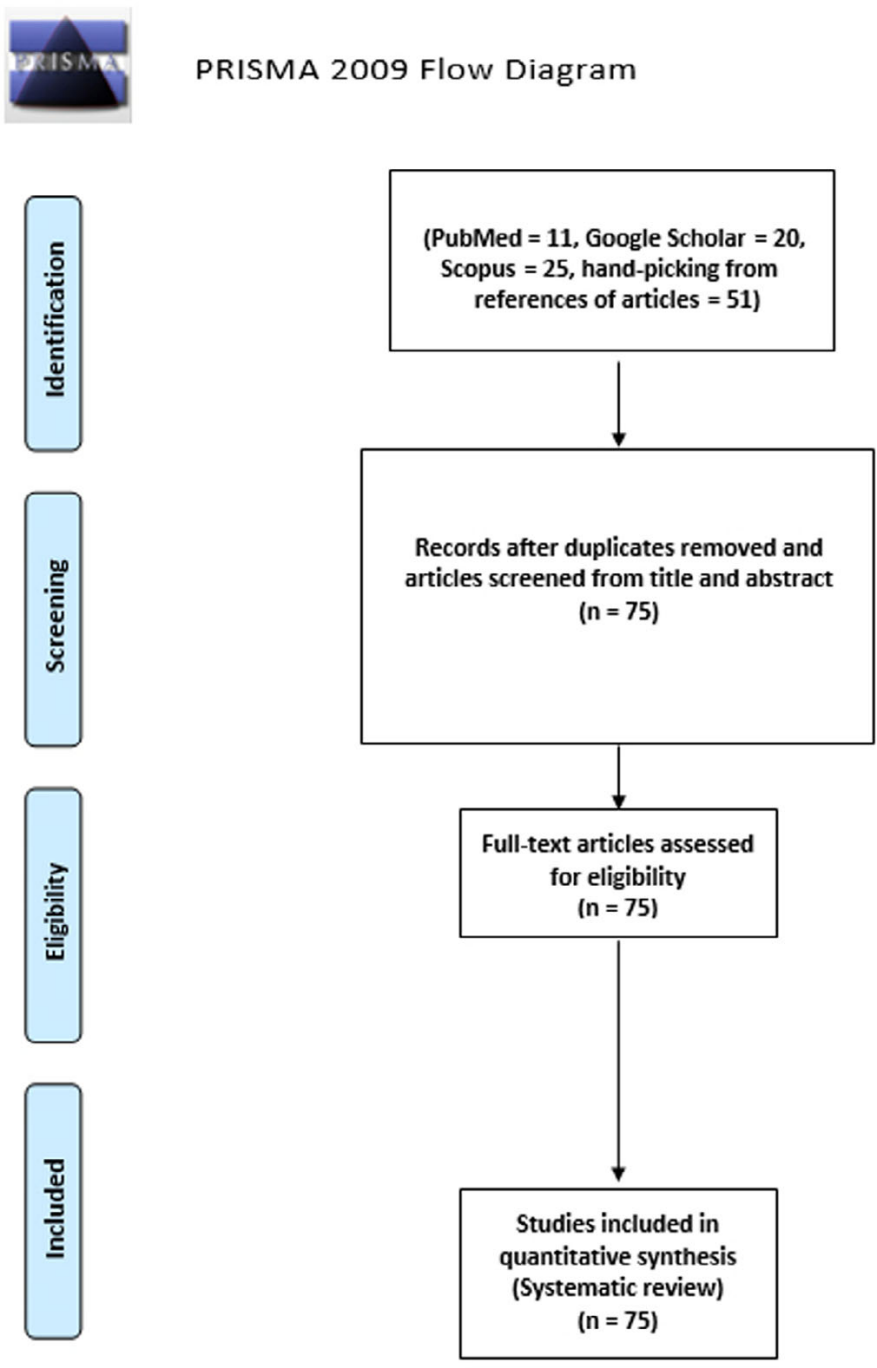
A PRISMA flowchart for the whole selection process

Two reviewers (FA and ES) will independently screen the studies, and any disagreements will be resolved by an independent screening of the disputed articles by a third reviewer (MY) who will be blinded to other reviewers' results. The results from each reviewer will be counter‐checked by the other reviewer to ensure the relevance of included studies. Initially, all the added articles will be screened by title, abstract, and keywords to remove irrelevant studies. Subsequently, a detailed screening will be performed by reading the full text of the studies to finalize articles for the review. Finalized articles will also be manually screened to detect eligible studies from the references. A PRISMA flowchart will be formulated for the whole selection process (Figure 1).

### 2.5 Data collection

Three reviewers (ES, HC, and FA) will divide the articles and extract the data from the finalized articles into the data extraction sheet. The results of each member will be counter‐checked by another member among the group to avoid errors during data collection.

The data collection sheet will comprise of a digital object identifier, author name, study title, year of publication, type of study, patient demographics (number of patients, gender, age, nationality, body mass index), age at diagnosis of BTM, method of diagnosis of BTM, treatment of BTM, age at diagnosis of EMH, symptoms at presentation with EMH, site of EMH, laboratory values at presentation (including complete blood count, Hgb electrophoresis, where available), imaging modalities used to diagnose EMH, histopathology details of EMH, management, and outcome.

### 2.6 Outcome measurement

The outcomes of the study will be divided into resolved (resolution of EMH), not resolved, progressed, and died.

### 2.7 Statistical analysis

The demographical and clinical parameters of the study population will be described via descriptive and summary statistics. All the continuous variables will be provided as means (±SD) or median (interquartile range) as required. Additionally, categorical variables were provided as numbers with percentages.

### 2.8 Assessment of methodological quality (risk of bias)

Two members (FA and ES) will assess the study's validity using the methodological methods and categories outlined in the Cochrane Collaboration Handbook, as well as PRISMA and other relevant guidelines. The GRADE assessment tool will be used to assess the quality of retrospective and prospective studies, whereas Joanna Briggs Institute (JBI) critical appraisal tools will be used to assess the quality of case reports and series.^14^, ^15^ If the members reach a disagreement, the third reviewer (MY), who will be blinded to other reviewers' results, will review the disputed studies to reach a conclusion.

## 3 DISCUSSION

EMH has been observed in various pathologies, including infection, malignancies, anemias (including beta‐thalassemia), and metabolic stress.^16^ In thalassemia, it is much more common in NTDT compared with TDT. In TDT, it is seen mainly in those patients in whom erythropoiesis is not adequately restricted by chronic transfusions.^7^ In one of the most extensive study cohorts of BTM patients with EMH, Ricchi et al compared the demographics and outcomes of patients with and without EMH.^8^ The authors found clinically and statistically significant differences in various parameters, including age at presentation, age at first transfusion, age when the regular transfusion was started, duration of regular transfusions, age at the initiation of chelation therapy, frequency of splenectomy, and serum ferritin levels among other parameters. The authors reported EMH mainly in the thoracic‐dorsal cord (98.8% of patients). In general, EMH is managed by hydroxycarbamide, transfusion therapy, radiotherapy, surgical intervention, or a combined management strategy can be applied.^7^

In our academic institute, we encountered two patients, in different timelines, with EMH in BTM. Both patients were managed by radiotherapy with excellent outcomes.^6^, ^10^ Our experience prompted us to explore the available literature on the management and outcomes of EMH in patients with TDT. The prevalence of EMH is quite variable, ranging from 1% to 13%.^8^ However, analysis of the limited retrospective studies and multiple case reports suggests that outcomes in patients managed for EMH in TDT are good. Although there is no agreed‐upon treatment guideline in such patients, various modalities have an excellent response, including conservative management such as blood transfusions and treatments such as radiotherapy and surgical decompression.^6^ This systematic review will focus on combining the reported prevalence, demographics, clinical course, management, and outcomes in these patients in an attempt to provide better evidence to formulate clear management guidelines.

## CONFLICT OF INTEREST

The authors declare that they have no competing interests.

## AUTHOR CONTRIBUTIONS

Conceptualization: Fateen Ata, Eihab A. Subahi, Mohamed A. Yassin

Investigation: Fateen Ata, Eihab A. Subahi, Hassan Choudry

Methodology: Fateen Ata, Eihab A. Subahi

Supervision: Mohamed A. Yassin

Writing ‐ Original Draft Preparation: Fateen Ata, Eihab A. Subahi, Hassan Choudry

Writing ‐ Review & Editing: Fateen Ata, Eihab A. Subahi, Hassan Choudry,, Mohamed A. Yassin

All authors have read and approved the final version of the manuscript.

Corresponding author or manuscript guarantor had full access to all of the data in this study and takes complete responsibility for the integrity of the data and the accuracy of the data analysis.

## TRANSPARENCY STATEMENT

The lead authors, Fateen Ata, Eihab A. Subahi, affirm that this manuscript is an honest, accurate, and transparent account of the study being reported; that no important aspects of the study have been omitted; and that any discrepancies from the study as planned (and, if relevant, registered) have been explained.

## ETHICS STATEMENT

Ethical approval is not required for this systematic review as only a secondary analysis of data already available in scientific databases will be conducted. The results of this review will be submitted for peer‐reviewed publication and will be presented at relevant conferences.

## Data Availability

Data sharing is not applicable.

## References

[1] Kanbour I , Chandra P , Soliman A , et al. Severe liver iron concentrations (LIC) in 24 patients with β‐thalassemia major: correlations with serum ferritin, liver enzymes and endocrine complications. Mediterr J Hematol Infect Dis. 2018;10:e2018062. 30416694 10.4084/MJHID.2018.062 PMC6223579

[2] De Sanctis V , Kattamis C , Canatan D , et al. β‐thalassemia distribution in the Old world: an ancient disease seen from a historical standpoint. Mediterr J Hematol Infect Dis. 2017;9:e2017018. 28293406 10.4084/MJHID.2017.018 PMC5333734

[3] Viprakasit V , Ekwattanakit S . Clinical classification, screening and diagnosis for thalassemia. Hematol Oncol Clin North Am. 2018;32:193‐211. 29458726 10.1016/j.hoc.2017.11.006

[4] Rund D , Rachmilewitz E . Beta‐thalassemia. N Engl J Med. 2005;353:1135‐1146. 16162884 10.1056/NEJMra050436

[5] Frangoul H , Altshuler D , Cappellini MD , et al. CRISPR‐Cas9 gene editing for sickle cell disease and β‐thalassemia. N Engl J Med. 2021;384:252‐260. 33283989 10.1056/NEJMoa2031054

[6] Subahi EA , Abdelrazek M , Yassin MA . Spinal cord compression due to extramedullary hematopoiesis in patient with Beta thalassemia major. Clin Case Rep. 2021;9:405‐409. 33489190 10.1002/ccr3.3542 PMC7812994

[7] Sousos N , Adamidou D , Klonizakis P , et al. Presence of the IVS‐I‐6‐mutated allele in beta‐thalassemia major patients correlates with extramedullary hematopoiesis incidence. Acta Haematol. 2017;137:175‐182. 28399542 10.1159/000463919

[8] Ricchi P , Meloni A , Spasiano A , et al. Extramedullary hematopoiesis is associated with lower cardiac iron loading in chronically transfused thalassemia patients. Am J Hematol. 2015;90:1008‐1012. 26228763 10.1002/ajh.24139

[9] Huang Y , Liu R , Wei X , et al. Erythropoiesis and iron homeostasis in non‐transfusion‐dependent thalassemia patients with extramedullary hematopoiesis. Biomed Res Int. 2019;2019:4504302. 30834265 10.1155/2019/4504302 PMC6374788

[10] Ahmad R , Okar L , Almasri H , et al. Low back pain in beta thalassemia major revealing sacral extramedullary hematopoeisis: a case report. Clin Case Rep. 2021;9(5):e04258. 34084519 10.1002/ccr3.4258 PMC8142798

[11] Stroup DF , Berlin JA , Morton SC , et al. Meta‐analysis of observational studies in epidemiology: a proposal for reporting. Meta‐analysis Of Observational Studies in Epidemiology (MOOSE) group. JAMA. 2000;283:2008‐2012. 10789670 10.1001/jama.283.15.2008

[12] Moher D , Liberati A , Tetzlaff J , Altman DG , PRISMA Group . Preferred reporting items for systematic reviews and meta‐analyses: the PRISMA statement. PLoS Med. 2009;6:e1000097. 19621072 10.1371/journal.pmed.1000097 PMC2707599

[13] Moher D , Shamseer L , Clarke M , et al; PRISMA‐P Group. Preferred reporting items for systematic review and meta‐analysis protocols (PRISMA‐P) 2015 statement. Syst Rev. 2015;4:1. 25554246 10.1186/2046-4053-4-1 PMC4320440

[14] Granholm A , Alhazzani W , Møller MH . Use of the GRADE approach in systematic reviews and guidelines. Br J Anaesth. 2019;123:554‐559. 31558313 10.1016/j.bja.2019.08.015

[15] Munn Z , Barker TH , Moola S , et al. Methodological quality of case series studies: an introduction to the JBI critical appraisal tool. JBI Database System Rev Implement Rep. 2019;18:2127‐2133. 10.11124/JBISRIR-D-19-00099 33038125

[16] Yang X , Chen D , Long H , Zhu B . The mechanisms of pathological extramedullary hematopoiesis in diseases. Cell Mol Life Sci. 2020;77:2723‐2738. 31974657 10.1007/s00018-020-03450-w PMC11104806

